# Supplementary material for: Application of SWATH Mass Spectrometry and Machine Learning in the Diagnosis of Inflammatory Bowel Disease Based on the Stool Proteome
Source: Biomedicines. 2024 Feb 1;12(2):333. doi: 10.3390/biomedicines12020333 (PMC10886680; doi:10.3390/biomedicines12020333)
Supplement: Supplementary file 1 [file biomedicines-12-00333-s001.zip › Figure S1.pdf]

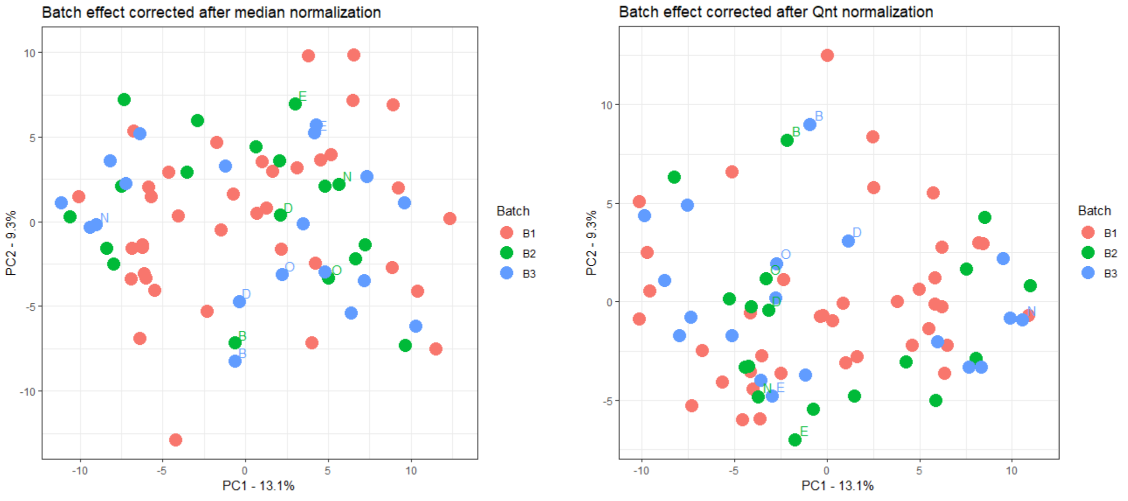

Figure S1 : The comparison examines the effects of two different normalization methods on batch effect correction. (a) PCA analysis of batch-corrected data after median normalization. (b) PCA analysis of batch-corrected data after quantile normalization. The comparison indicates no significant differences among them.
