# Supplementary material for: Application of SWATH Mass Spectrometry and Machine Learning in the Diagnosis of Inflammatory Bowel Disease Based on the Stool Proteome
Source: Biomedicines. 2024 Feb 1;12(2):333. doi: 10.3390/biomedicines12020333 (PMC10886680; doi:10.3390/biomedicines12020333)
Supplement: Supplementary file 1 [file biomedicines-12-00333-s001.zip › Figure S2.pdf]

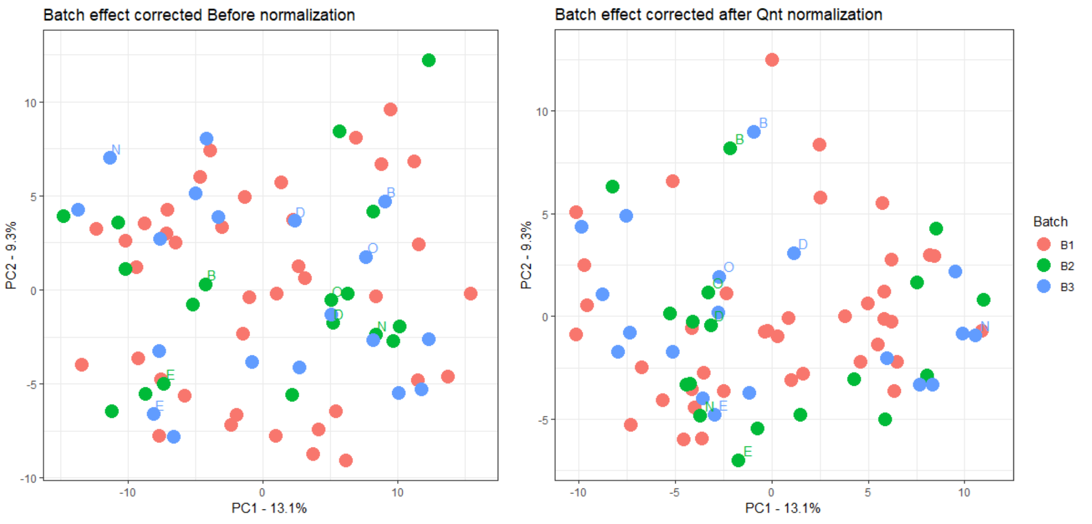

Figure S2 : The comparison examines the effect of normalization before and after batch effect correction. (a) PCA analysis of batch effect corrected data followed by quantile normalization. (b) PCA analysis of batch-corrected data after quantile normalization. The comparison indicates no significant differences among them.
